# A comparative study on bone density before and after implant placement using osseodensification technique: a clinical evaluation

**DOI:** 10.1186/s40729-024-00565-8

**Published:** 2024-11-19

**Authors:** Sunil Kumar Vaddamanu, Ravinder S. Saini, Rajesh Vyas, Masroor Ahmed Kanji, Abdulkhaliq Ali F. Alshadidi, Salah Hafedh, Marco Cicciù, Giuseppe Minervini

**Affiliations:** 1 Department of Allied Dental Health Sciences, College of Applied Medical Sciences, King Khalid University, Abha, Saudi Arabia; 2 Orthodontics Department, Faculty of Dentistry, Sana’a University, Sana’a, Yemen; 3 Department of Biomedical and Surgical and Biomedical Sciences, Catania University, Catania, Italy; 4 Saveetha Institute of Medical and Technical Sciences (SIMATS), Saveetha Dental College and Hospitals, Saveetha University, Chennai, India; 5 Multidisciplinary Department of Medical-Surgical and Odontostomatological Specialties, University of Campania “Luigi Vanvitelli”, Naples, Italy

**Keywords:** Osseodensification, Dental implants, Bone density, Osteotomy preparation, Primary stability, Autografting

## Abstract

**Background:**

Dental implant success critically depends on the primary stability of the implant, which is significantly influenced by the bone density at the osteotomy site. Traditional drilling techniques for osteotomy preparation often compromise bone volume and quality. This study aimed to evaluate the impact of osseodensification, a novel osteotomy preparation technique, on bone density and implant stability. The technique utilizes specialized drills that operate in a counter-clockwise direction to compact autografted bone laterally and apically, preserving and enhancing bone density.

**Methods:**

A total of 32 patients undergoing dental implant surgery were included in this study. Pre-operative and post-operative bone densities at the apical, mesial, and distal regions of the osteotomy sites were measured using Dentascan (CT) and analyzed with Radiant DICOM software. The study utilized osseodensification drills for osteotomy preparation, comparing pre-operative and post-operative bone densities to assess the technique’s efficacy.

**Results:**

The study found a statistically significant increase in bone density post-operatively (*p* < 0.001), with the greatest improvement observed in the distal region, followed by the mesial and apical regions. The findings underscore osseodensification’s effectiveness in enhancing bone density and primary stability, with the distal region exhibiting the highest bone density.

**Conclusion:**

Osseodensification represents a significant advancement in implant dentistry for osteotomy preparation. By preserving and increasing bone density through compact autografting, this technique not only improves primary stability but also offers potential benefits in indirect sinus lifting and alveolar ridge expansion. The study advocates for the broader adoption of osseodensification drills in clinical practice to achieve better outcomes in dental implantology.

**Trial Registration:**

This study received ethical approval from The Research Ethics Committee at King Khalid University’s under Approval no. ECM#2024 − 216. Additionally, it was registered with ClinicalTrials.gov, identifier no: NCT06268639.

**Graphical Abstract:**

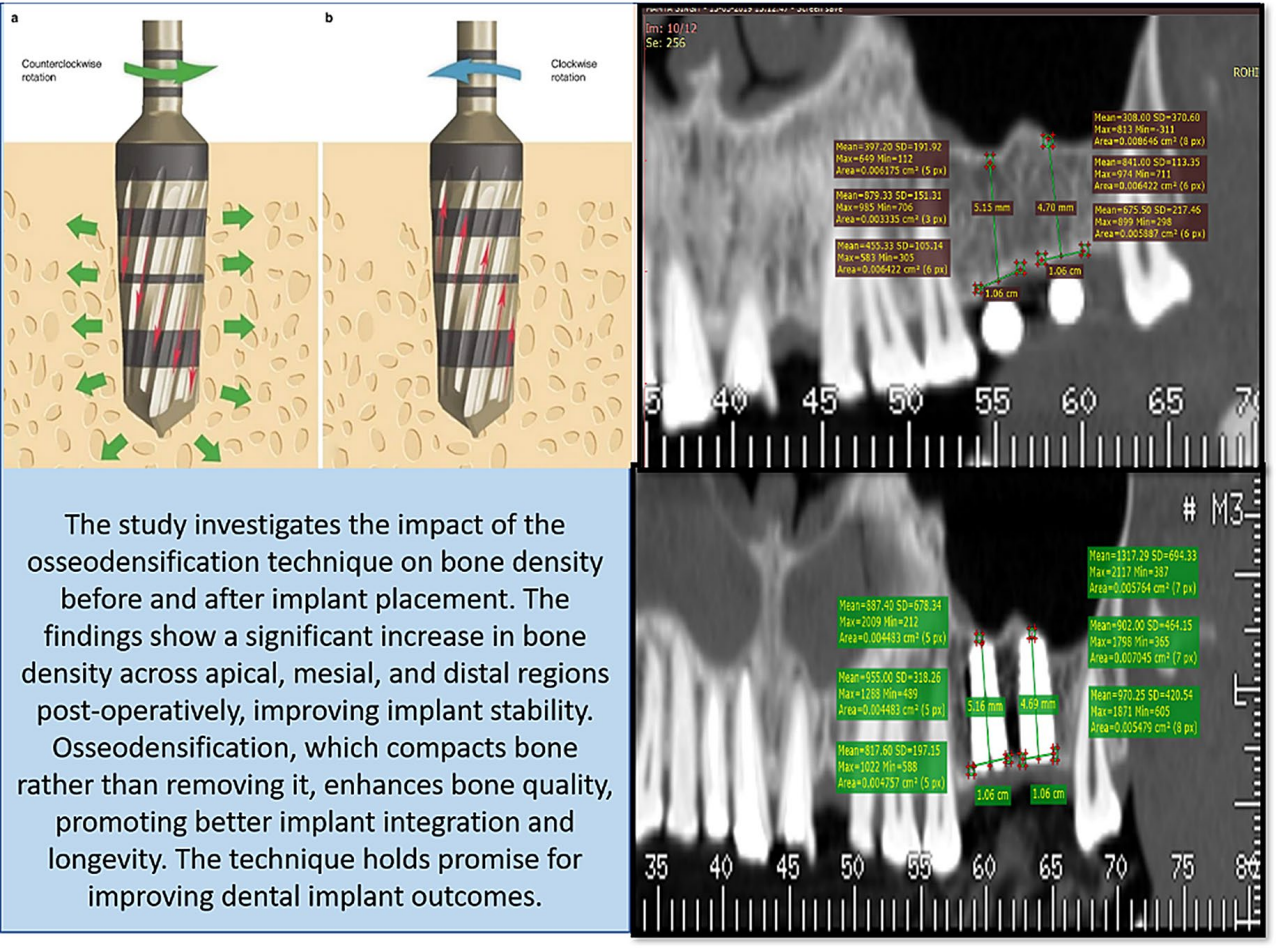

## Background

The increasing adoption of dental implants as a preferred modality for replacing missing teeth is underscored by the critical role of implant therapy success, which hinges on the stability of the primary implant to facilitate osseointegration [1, 2]. Osseointegration, as defined by Brånemark and colleagues in 1977, is a direct structural and functional connection between living bone and the surface of the implant [2]. This phenomenon is pivotal for the implant’s loading capacity and its long-term clinical success. The achievement of primary stability, influenced by variables such as surgical techniques, and the quantity and quality of bone, is deemed essential for optimal osseointegration [3–5].

The literature highlights the multifaceted aspects contributing to osseointegration, including the material, surface, and design of the implant, host factors, surgical techniques, and biomechanical preparation [4]. Central to the placement of dental implants, achieving primary stability is governed by bone density, surgical protocol, and the macro and micro-design of the implant, among others. The conventional approach to creating osteotomies involves bone-removal drills, with the final drill size being slightly smaller than the implant to ensure primary stability. This practice aims to preserve bone bulk, thereby enhancing the primary mechanical stability of bone-to-implant contact and, subsequently, the implant’s secondary stability [5–8].

Expanding on these findings, previous studies have further validated the impact of bone quality on implant stability, highlighting the benefits of adapted surgical techniques in areas of low bone density to improve implant survival rates [9, 10].

Earlier in implant dentistry the surgical technique used were undersized implant site preparation [10] and osteotome was used to condense the bone [11]. The use of the osteotome in poor density bone allows fracturing and condensing of bone trabeculae, but this technique does not improve peri-implant bone density and it may also cause bone fracture [12].

In contrast to traditional drilling techniques, which remove bone to create osteotomies, the advent of osseodensification, a technique developed by Huwais in 2013, presents a novel approach. This technique, characterized by the use of specially designed burs that compact and autograft bone along the osteotomy, not only preserves bone bulk but also enhances the bone density around dental implants, thereby improving their primary stability. Unlike conventional drilling, osseodensification does not excavate bone tissue. Rather, it preserves bone bulk, so bone tissue is simultaneously compacted and autografted in an outwardly expanding direction to form the osteotomy. The unexcavated bone mass increases the bone dentistry in both the mesial-distal and apical osteotomy site [1, 13, 14]. That increase in bone density is checked by the comparing the patient pre-operative and post-operative dentascan (CT) [15].

Osseodensification represents a modern biomechanical bone preparation process that facilitates the placement of dental implants by developing a densification layer around the osteotomy site [15–19]. This process, which involves compacting and autografting bone across the entire depth of the osteotomy, has been shown to increase bone density and expansion, thereby offering a promising alternative to traditional drilling techniques.

Subsequent research, has continued to explore the effects of bone density and surgical techniques on implant stability and success [20–24]. These investigations have contributed to a nuanced understanding of how modifications in surgical approach, such as osseodensification, can positively influence implant outcomes, especially in low-density bone environments.

The cumulative evidence from these studies underscores a critical evolution in dental implantology. By focusing on the interplay between bone density, surgical techniques, and implant design, the field has moved towards personalized, evidence-based strategies that enhance implant stability and success.

This study aims to elucidate the impact of pre-operative and post-operative increases in bone density around implant surfaces facilitated by osseodensification drills. By employing digital radiographic techniques such as dentascan (CT), this investigation seeks to delineate the efficacy of osseodensification drills in achieving enhanced primary stability and to document the variation in bone density pre and post-operatively.

### Objectives

1. **To evaluate preoperative bone density**: This objective involves the comprehensive assessment of the bone density at the intended site of implant placement prior to the surgical procedure. Such an evaluation is crucial for establishing a baseline against which postoperative outcomes can be compared, thereby enabling an understanding of the bone’s condition before any intervention.
2. **To evaluate postoperative bone density using osseodensification technique**: Postoperative assessment of bone density is essential to ascertain the effectiveness of the osseodensification technique. By comparing the bone density after the implant placement with the preoperative levels, this objective seeks to quantify the impact of osseodensification on enhancing bone density around the implant site.
3. **To inter-compare bone density before and after placement using osseodensification technique**: The core of the study revolves around a comparative analysis of bone density measurements taken before and after the application of the osseodensification technique. This comparative analysis aims to provide empirical evidence regarding the technique’s role in improving bone density, which is a critical factor in achieving primary stability and long-term success of dental implants.

The realization of these objectives will contribute significantly to the body of knowledge on dental implantology, specifically highlighting the benefits and implications of employing osseodensification as a technique for preparing the implant site. Through this study, dental professionals and researchers will gain valuable insights into advanced surgical techniques that could potentially enhance the success rates of dental implant procedures.

## Methods

This study received ethical approval from The Research Ethics Committee at King Khalid University’s under Approval no. ECM#2024 − 216. Additionally, it was registered with ClinicalTrials.gov, identifier no: NCT06268639.

### Inclusion criteria

The study focused on individuals exhibiting either partial or complete edentulous ridges in the maxilla or mandible. This broad inclusion criterion was chosen to encompass a wide range of dental implant candidates, providing a comprehensive understanding of osseodensification’s impact across varied clinical scenarios.

### Exclusion criteria

Individuals were excluded from the study under the following conditions:

- **Fractured jaw**: Patients with a history of jaw fractures were excluded to prevent confounding factors related to bone healing and integrity that could potentially skew the results of the study regarding the effectiveness of osseodensification.
- **General bone or blood disorders**: Those with systemic bone or blood disorders that could impair osseointegration were also excluded. Conditions such as osteoporosis, osteopenia, or hematologic disorders could interfere with the body’s ability to form a stable integration between the implant and bone, thus affecting the study’s outcomes.

These criteria were meticulously set to ensure the study’s participants were best suited to provide clear insights into the effectiveness of osseodensification in enhancing bone density at the implant site, thereby offering a focused examination of its potential benefits in dental implantology.

The methodology of this study encompasses a detailed procedural framework, systematically designed to evaluate the impact of osseodensification on bone density at dental implant sites. This methodology is structured into several key stages (Table 1).

**Table 1:** Comparison of preoperative and postoperative bone density

Site	Pre-op mean density (HU)	Post-op mean density (HU)	Change in density (HU)	*p*-value
Apical	821.3 ± 485.5	1126.7 ± 373.6	305.5	< 0.001*
Mesial	758.3 ± 268.3	1203.7 ± 323.2	445.4	< 0.001*
Distal	781.4 ± 283.6	1238.4 ± 332.7	457.1	< 0.001*

*The p-value indicates that the increase in bone density is statistically significant for each site

### Selection of the sample size

Utilizing G*Power software, the study defined a sample size of 32 patients based on specific statistical parameters: a significance level (α) of 0.05 and a power of 0.80 (80%). This population was divided into two groups for analytical purposes:

- **Group I**: Consisting of the pre-operative bone density at 32 osteotomy sites.
- **Group II**: Comprising the post-operative bone density at the same 32 osteotomy sites.

### Fabrication of splint with radio-opaque marker

The initial patient appointment involved taking a diagnostic impression using irreversible hydrocolloid material, which was then poured into a dental stone using a vacuum mixing machine. A surgical splint was fabricated from auto-polymerizing acrylic resin on the diagnostic cast, incorporating Gutta-percha as a radio-opaque marker at the planned implant sites. This splint, finished and polished, was used during the Dentascan (CT) to accurately indicate osteotomy locations.

### Pre-operative bone density analysis

**Fig. 1:**
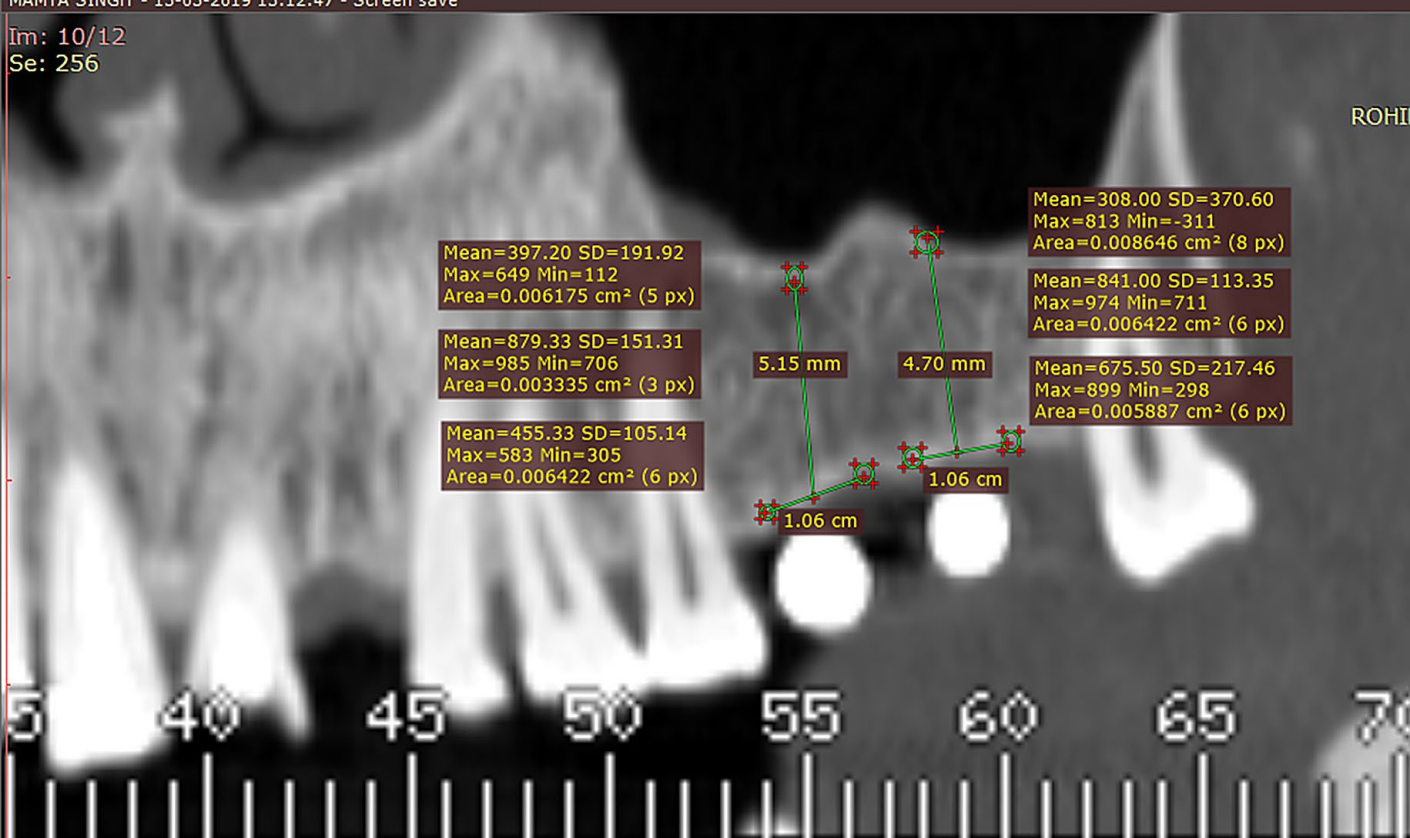
Showing pre-operative bone density analysis

The second patient appointment entailed a preoperative Dentascan (CT)( GE Healthcare LightSpeed. Exposure 90kv, 2.5 mA,15s and dose – 1179mGy.cm^2^) at Rohilkhand Medical College and Hospital’s Department of Radiology. Using Radiant DICOM software, the preoperative scans, marked with radio-opaque indicators for precise osteotomy positioning, were analyzed. Bone density was measured in Hounsfield units at apical, mesial, and distal points relative to each implant site, with the data recorded and categorized as Group I. Figure 1.

### Osteotomy site preparation

**Fig. 2:**
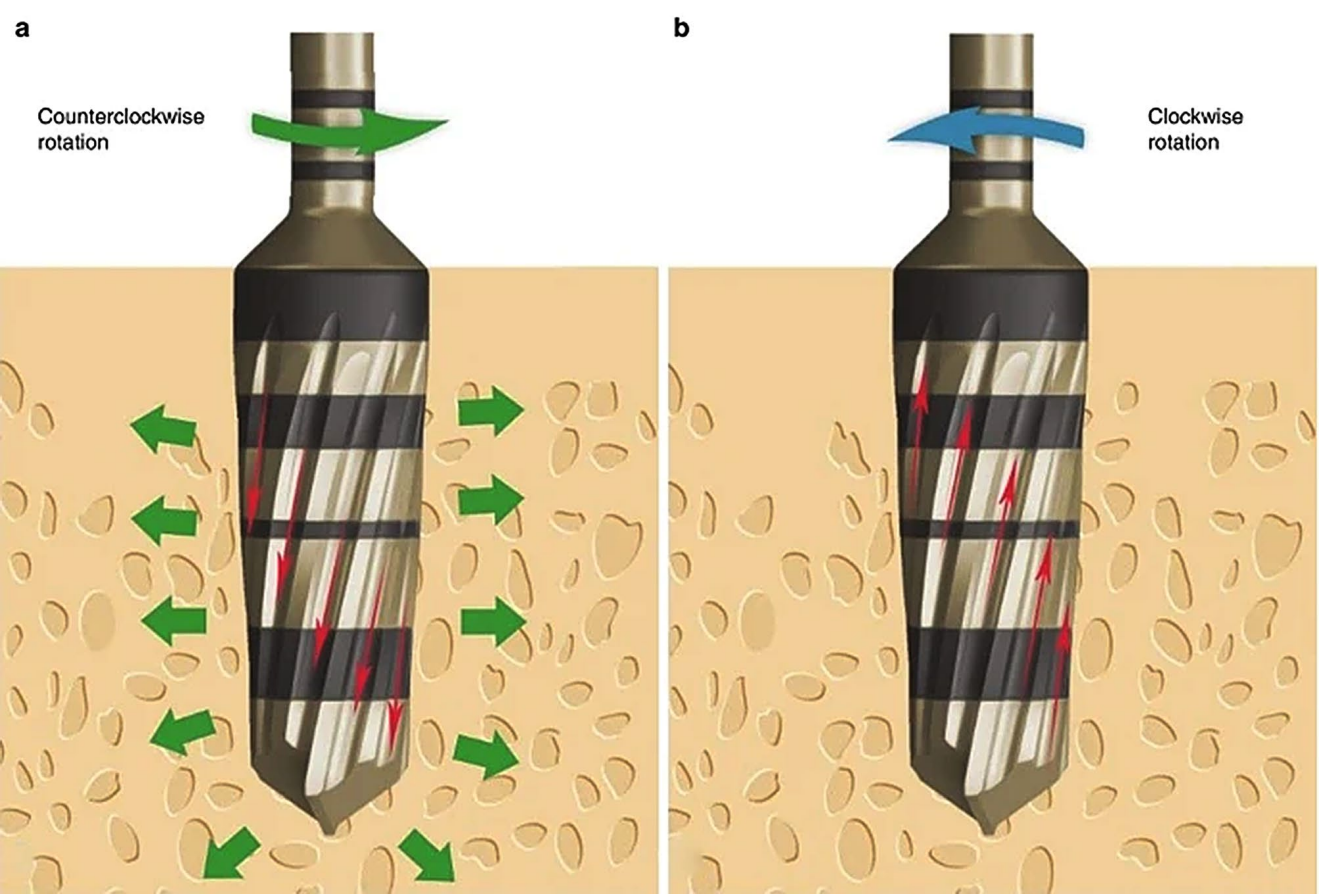
**a**) graphical representation of osseodenstification technique **b**) graphical representation of implant site preparation Courtesy by – Osseodensification Paraigm Shift [25]

Under local anesthesia, the surgery proceeded in the Department of Prosthodontics. After administering an incision and raising a flap, a pilot osteotomy was created using a pilot drill at speeds between 800 and 1500 RPM. Subsequent osseodensification drilling expanded the osteotomy sites, followed by implant placement, flap closure, and suturing [25]. Figure 2 Post-operative care was administered, and patients were prepared for subsequent Dentascan analysis.

### Post-operative bone density analysis

**Fig. 3:**
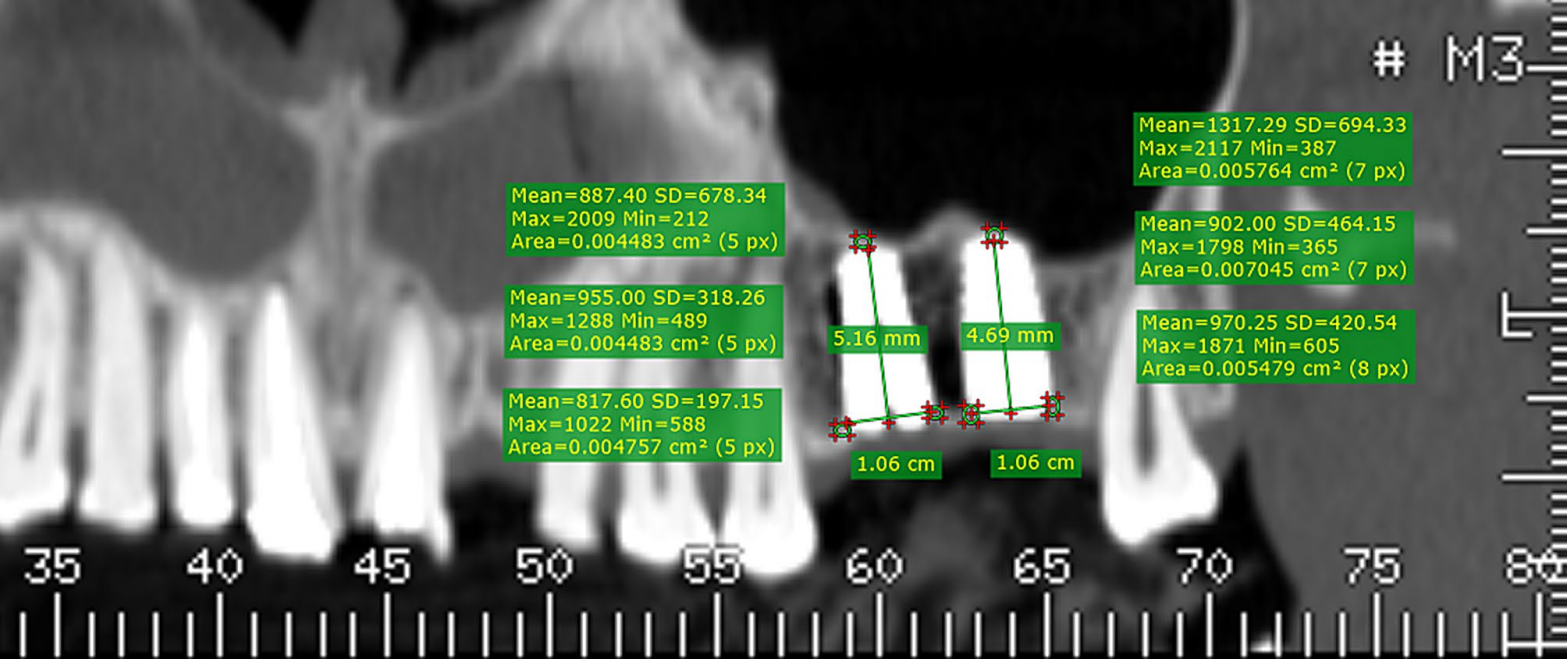
Showing post-operative bone density analysis

Post-operative Dentascan evaluations were conducted, again using Radiant DICOM software to assess bone density at specified positions around the implant sites, with results recorded in Hounsfield units and categorized as Group II. Figure 3.

### Statistical analysis

Data collected were organized using Microsoft Excel and analyzed with SPSS version 22. The analysis focused on mean bone density and standard deviation, employing the student paired t-test to identify significant differences in bone density from pre-operative to post-operative stages. A P-value less than 0.05 was deemed indicative of statistical significance.

## Results

The study meticulously evaluated the impact of the osseodensification technique on bone density at dental implant sites, comparing pre-operative and post-operative values across apical, mesial, and distal regions. The findings reveal a notable increase in bone density following the application of osseodensification, indicating its effectiveness in enhancing the bone environment for dental implants. Below is a consolidated presentation of the results:

### Overview of bone density changes

#### Apical region

- **Pre-operative mean bone density**: 821.3 ± 485.5 Hounsfield Units (HU).
- **Post-operative mean bone density**: 1126.7 ± 373.6 HU.
- **Increase in mean bone density**: 305.4 HU.
- The analysis showed a significant increase in bone density, with values ranging from 247 HU to 2860 HU pre-operatively and from 420 HU to 2143 HU post-operatively.

#### Mesial region

- **Pre-operative mean bone density**: 758.3 ± 268.3 HU.
- **Post-operative mean bone density**: 1203.7 ± 323.2 HU.
- **Increase in mean bone density**: 445.4 HU.
- Bone density improvements were observed, with pre-operative values from 225 HU to 1497 HU and post-operative values from 679 HU to 1908 HU.

#### Distal region

- **Pre-operative mean bone density**: 781.4 ± 283.6 HU.
- **Post-operative mean bone density**: 1238.4 ± 332.7 HU.
- **Increase in mean bone density**: 457.1 HU.
- Significant gains in bone density were noted, with pre-operative values from 204 HU to 1509 HU and post-operative values from 552 HU to 1879 HU.

### Significance of findings

The study’s findings demonstrate a significant enhancement in bone density across all regions analyzed (apical, mesial, and distal) following the use of osseodensification. This technique not only preserves but also significantly improves the density of bone around dental implants. Such an increase in bone quality is critical for achieving primary stability, which is essential for the long-term success of dental implants.

The observed increases in bone density, evidenced by the substantial changes in Hounsfield Units, underscore the effectiveness of osseodensification as a valuable technique in dental implantology. By promoting a more favorable bone environment for implant integration, osseodensification could lead to improved outcomes for dental implants, particularly in cases where bone quality is a concern (Table 2).

Overall, the results affirm the hypothesis that osseodensification is a beneficial adjunct to traditional dental implant preparation techniques, offering a promising avenue for enhancing osseointegration and potentially increasing the success rates of dental implants. The increase in bone density from pre-operative to post-operative measurements was statistically significant (*p* < 0.001). The significant differences in bone density from pre-operative to post-operative phases validate the technique’s role in improving the structural and functional integration of dental implants with the surrounding bone.

**Table 2:** Preoperative and postoperative density ranges

Site	Pre-op min (HU)	Pre-op max (HU)	Post-op min (HU)	Post-op max (HU)
Apical	247	2860	420	2143
Mesial	225	1497	679	1908
Distal	204	1509	552	1879

## Discussion

The field of implant dentistry has experienced significant advancements in recent decades, with increasing patient awareness about the latest technological and surgical improvements. This evolution has positioned implant dentistry as the preferred treatment option for both completely and partially edentulous patients, utilizing a variety of implant modules that leverage both conventional and computerized imaging technologies for optimal implant placement. A critical factor in the success of implant therapy is the achievement of primary stability, which is closely linked to the bone density of the patient [26]. Consequently, primary stability tends to be higher in the mandibular arch than in the maxillary arch, with bone density, surgical protocol, implant thread type, and geometry being key factors in enhancing implant primary stability [8, 9, 27].

Historically, surgical techniques in implant dentistry have included undersized implant site preparation and the use of osteotomes for bone condensation [10, 11]. However, these methods, particularly in low-density bone, may not improve peri-implant bone density and risk bone fracture [12].

This study explores the enhancement of bone density at various osteotomy sites using the osseodensification drill, introduced by Dr. Salah Huwais in 2014. Unlike traditional drilling, osseodensification preserves bone bulk by compacting and autografting bone in an outwardly expanding direction, thus increasing bone density around the implant. This increase in bone density is quantitatively assessed through pre-operative and post-operative Dentascan (CT) comparisons [1, 2, 15, 28–30].

Radiographic guides, essential for accurate treatment planning in implant dentistry, were fabricated using various radiopaque markers, including gutta-percha, to pinpoint the osteotomy sites on Dentascan (CT). These guides not only facilitate precise implant placement but also allow for the assessment of surgical implant sites in three dimensions, a significant advancement over traditional two-dimensional radiographic techniques [17].

The study utilized the Radiant DICOM software for pre-operative and post-operative bone density analysis, highlighting the software’s capabilities in image manipulation and measurement [31–33]. Following the osseodensification technique, a significant increase in bone density was observed, particularly in the mesial and distal positions relative to the apical position. This finding is consistent with previous research indicating that osseodensification not only enhances primary stability but also promotes bone healing and improves the prognosis of the implant.

Osseodensification, by preventing bone excavation and facilitating lateral and apical bone compaction, represents a significant advancement in implant bed preparation. This technique offers numerous advantages, including enhanced bone density, residual ridge expansion, and increased implant stability, thereby contributing to the long-term success of dental implants. This study underscores the importance of osseodensification in improving peri-implant bone density and primary stability, marking a pivotal development in implant dentistry.

## Conclusion

The findings from this study demonstrate that osseodensification, through the use of specialized drills in a counter-clockwise direction, significantly enhances bone density at the osteotomy site, particularly in the distal direction where the greatest bone density is observed, followed by the mesial and then the apical areas. This densification process not only preserves but also augments bone volume and quality by compacting autografted bone laterally and apically, thereby improving primary stability and facilitating bone healing around the implant. The osseodensification technique emerges as a groundbreaking, bone-preserving method for osteotomy preparation, offering additional benefits such as potential indirect sinus lifting and alveolar ridge expansion. This technique represents a significant advancement in implant dentistry, promoting better outcomes for implant stability and longevity.

## Data Availability

Dr. Sunil Kumar Vaddamanu will have access to the data that were the basis for this article, and can be reached out for data in case is needed for review.
